# Healthcare resource use and costs of multiple sclerosis patients in Germany before and during fampridine treatment

**DOI:** 10.1186/s12883-017-0844-z

**Published:** 2017-03-27

**Authors:** Tjalf Ziemssen, Christine Prosser, Jennifer Scarlet Haas, Andrew Lee, Sebastian Braun, Pamela Landsman-Blumberg, Angela Kempel, Erika Gleißner, Sarita Patel, Ming-Yi Huang

**Affiliations:** 1 Universitätsklinium Dresden, Fetscherstraße 74, 01307 Dresden, Germany; 2 Xcenda GmbH, Lange Laube 31, D-30159 Hannover, Germany; 3 Biogen, Cambridge, MA USA; 4 Xcenda LLC, Palm Harbor, FL USA; 5 Biogen GmbH, Carl-Zeiss-Ring 6, 85737 Ismaning, Germany

**Keywords:** Multiple sclerosis, Claims data, Germany, Fampridine

## Abstract

**Background:**

Multiple sclerosis (MS) patients often suffer from gait impairment and fampridine is indicated to medically improve walking ability in this population. Patient characteristics, healthcare resource use, and costs of MS patients on fampridine treatment for 12 months in Germany were analyzed.

**Methods:**

A retrospective claims database analysis was conducted including MS patients who initiated fampridine treatment (index date) between July 2011 and December 2013. Continuous insurance enrollment during 12 months pre- and post-index date was required, as was at least 1 additional fampridine prescription in the fourth quarter after the index date. Patient characteristics were evaluated and pre- vs post-index MS-related healthcare utilization and costs were compared.

**Results:**

A total of 562 patients were included in this study. The mean (standard deviation [SD]) age was 50.5 (9.8) years and 63% were female. In the treatment period, almost every patient had at least 1 MS-related outpatient visit, 24% were hospitalized due to MS, and 79% utilized MS-specific physical therapy in addition to the fampridine treatment. Total MS-related healthcare costs were significantly higher in the fampridine treatment period than in the period prior to fampridine initiation (€17,392 vs €10,960, *P* < 0.001). While this difference was driven primarily by prescription costs, MS-related inpatient costs were lower during fampridine treatment (€1,333 vs €1,565, *P* < 0.001).

**Conclusions:**

Physical therapy is mainly used concomitant to fampridine treatment. While healthcare costs were higher during fampridine treatment compared to the pre-treatment period, inpatient costs were lower. Further research is necessary to better understand the fampridine influence.

**Electronic supplementary material:**

The online version of this article (doi:10.1186/s12883-017-0844-z) contains supplementary material, which is available to authorized users.

## Background

Multiple sclerosis (MS) is a chronic and progressive autoimmune disease of the central nervous system. MS patients suffer from diverse symptoms, whereas gait disturbance is one of the major problems that occurs frequently [1–3]. An estimated 40 to 90% of patients with MS experience walking impairment [1, 4, 5]. Fampridine is the first and only available medical treatment for improving walking ability in patients with MS and it has been licensed since 07/2011 in Europe [6]. The fampridine tablets (10 mg) are given twice a day, and if no improvement is shown after 2 weeks, the treatment should be stopped [6].

Due to its relative novelty, no information on fampridine-treated patients under real-life conditions is available in Germany. This information can contribute to understanding the unmet needs of this patient group. Furthermore, limited data assessing the resource implications of treating MS mobility symptoms are available.

This study aims at identifying the treatment, patient characteristics, MS-related healthcare resource use, and costs of patients staying on fampridine therapy for 1 year after treatment initiation. Furthermore, a comparison of the MS-specific healthcare resource use and costs during fampridine treatment with the pre-treatment period without fampridine was also conducted.

## Methods

This retrospective claims data analysis was conducted using data from the Health Risk Institute (HRI) research database.

### Database

The HRI research database comprises claims data from 75 of the 120 statutory health insurances in Germany. The analysis sample includes the utilization and costs of services for approximately 4 million covered lives through 2014 on an anonymized, individual level. This sample represents 4.8% of the population in Germany and is already adjusted for age and gender for the German population. Furthermore, the HRI research database is considered to have good external validity to the German population in terms of morbidity, mortality, and drug use [7].

### Patient selection

All adult patients initiating treatment with fampridine between July 2011 and December 2013 in the database were identified, and the first prescription fill of fampridine (Anatomical Therapeutic Chemical code N07XX07) in this period determined the index quarter. Patients were included if they were continuously enrolled 4 quarters before and 4 quarters after the index quarter. At least 1 MS diagnosis (International Classification of Diseases, 10th Revision, German Modification [ICD-10-GM] G35.XX) in the inpatient sector (main or secondary diagnosis) or in the outpatient sector (verified diagnosis) during the index quarter or the preceding quarters was required. Furthermore, at least 1 additional fampridine prescription fill in the fourth quarter after the index served as a proxy indicating continuous fampridine treatment within the post-index period.

The identified patients were then stratified by DMT use, age and by use of antispasmodics to identify differences related to specific patient characteristics.

The full study population was stratified according to their disease-modifying therapy (DMT) use during the study period, defined as “continuous DMT”, “discontinuous DMT” and “no DMT”. This stratification was performed to isolate the effect of fampridine from possible effects of DMT treatment. The included DMTs were intramuscular (IM) interferon (INF) beta-1a, subcutaneous (SC) INF beta-1a, INF beta-1b, glatiramer acetate, natalizumab, teriflunomide, dimethyl fumarate, and fingolimod. Continuous DMT users were required to have at least 1 prescription claim for a DMT in the fourth quarter before the index quarter, 1 in the index quarter itself, and 1 in the fourth quarter after the index quarter. Switches between the DMTs were not permitted in this subgroup. The discontinuous DMT cohort was defined as having a prescription claim for at least 1 DMT in any of the 9 quarters (4 quarters pre-index, index quarter, and 4 quarters post-index) where DMT switches were allowed. The subgroup of patients with no DMT had no prescription claims for any DMT in any of the 9 study quarters.

The second stratification divided the study population by age, including the subgroups aged 18 to 49 years and ≥50 years of age.

For the third stratification, all fampridine patients were subdivided into users and non-users of antispasmodic treatment. Users were defined as having at least 1 prescription of an antispasmodic treatment (baclofen, botulinum toxin, dantrolene, tizanidine, tolperisone, tetrazepam, gabapentin, cannabinoids) anytime during the 24-month observation period. Non-users had no evidence of symptomatic treatment within the study period.

### Outcomes

Patient characteristics, including demographics, co-medication use (including DMTs and other MS-related medications using Anatomical therapeutic chemical classification system [ATC] codes), and comorbidities measured with the Charlson Comorbidity Index (CCI), and the most frequent diagnoses (top 10) in the 4 quarters before the index fampridine prescription were assessed.

The outcomes consisted of MS-related healthcare resource use for the inpatient, outpatient, and pharmacotherapy sectors. For the inpatient stays, MS-specific hospital visits were those with the MS ICD-10-GM code G35.XX as the primary diagnosis. Outpatient diagnoses were coded by different physician specialties, including but not limited to: general practitioners, neurologists, emergency physicians, and internists. The diagnoses are only coded on a quarterly basis and not directly linked to an intervention in the German healthcare system; therefore, an approximation of MS-related outpatient visits was assessed by calculating the number of visits with an MS ICD-10-GM diagnosis code in the same quarter. The same method was applied for the physical therapy visits. Furthermore, corticosteroid prescription fills, MS-related sick leave days (with a MS ICD-10-GM diagnosis code), and prescriptions for mobility-related devices were also assessed (eg, wheelchair, cane, etc.).

The MS-related healthcare costs in Euros were calculated using the costs for the use of resources described above. Pharmacotherapy costs included the corticosteroid prescriptions, DMTs, fampridine, and other MS-related medications, including antidementia; antidepressants; antiepileptic; urinary antispasmodics; selected muscle relaxants such as baclofen, botulinum toxin, dantrolene, tizanidine, tolperisone, and tetrazepam; selected medications to manage fatigue such as amantadine and modafinil; selected drugs for sexual dysfunction such as sildenafil, tadalafil, and tibolone; selected drugs against tremor such as propranolol; as well as benzodiazepine, and cannabinoids, according to Deutsche Gesellschaft für Neurologie (German Neurological Society) [8], Hoer et al. [9], and Bonafede et al. [10] The costs were then adjusted for inflation for the year 2014 using the general rate of inflation for Germany [11].

Baseline patient characteristics were assessed in the pre-index period. Healthcare utilization and costs for the 1-year treatment period were analyzed using descriptive statistics. Baseline characteristics, healthcare resource use, and costs were also stratified by the subgroups previously noted. Mean change (pre – post) and SD were computed for continuous healthcare resource use and cost measures. One-sample *t*-tests or Wilcoxon signed-rank tests were used for the evaluation of change measures (pre – post), depending on the distributional properties of the measure under evaluation. A *P*-value <0.05 denoted statistical significance and the statistical software SAS version 9.2 (SAS Institute, Cary NC, USA) was used for all analyses.

## Results

### Patient characteristics

Out of 1318 identified patients treated with fampridine, 43% (*N* = 562) met all study criteria. Most of the patients were excluded because they did not have a fampridine prescription fill in the 4^th^ quarter after the index quarter. The mean age was 50.5 years and 63% were female. The most frequently prescribed medications in the pre-index period were muscle relaxants with 40.4% (such as baclofen with 26.2%) and antidepressants (31.9%) (see Table 1). On average, fampridine was prescribed 11 times per patient in the 12-month post-index period (SD 3.4).

**Table 1:** Patient characteristics

Characteristic	*N* = 562
Age in years, mean (SD)	50.5 (9.8)
Median	50.5
Minimum, maximum	23.7, 79.2
Age group, n (%)	
18–34	30 (5.3%)
35–44	128 (22.8%)
45–54	229 (40.7%)
55–64	137 (24.4%)
65+	38 (6.8%)
Female, n (%)	352 (62.6%)
Index year, n (%)	
2011	185 (32.9%)
2012	265 (47.2%)
2013	112 (19.9%)
MS ICD-10-GM codes at index quarter, n (%)^a^	
G35.0: Initial manifestation of MS	92 (16.4%)
G35.1: Mainly relapsing/remitting MS	288 (51.2%)
G35.2: Primary progressive MS	121 (21.5%)
G35.3: Secondary progressive MS	175 (31.1%)
G35.9: MS, unspecified	450 (80.1%)
Exclusively unspecified diagnosis (G35.9)	85 (15.1%)
First prescribed DMT, n (%)^c^	
IM INF beta-1a	50 (8.9%)
SC INF beta-1a	48 (8.5%)
SC INF beta-1b	59 (10.5%)
Glatiramer acetate	85 (15.1%)
Natalizumab	41 (7.3%)
Teriflunomide	0 (0%)
Fingolimod	19 (3.4%)
Dimethyl fumarate	3 (0.5%)
None	257 (45.7%)
MS-related medications, n (%)^d^	
Corticosteroids	225 (40.0%)
Immunosuppressants	84 (14.9%)
Drugs for symptom relief, n (%)^d^	
Antidementia	6 (1.1%)
Antidepressants	179 (31.9%)
Antiepileptics	97 (17.3%)
Select muscle relaxants	227 (40.4%)
Urinary antispasmodics	122 (21.7%)
Medications to manage fatigue	37 (6.6%)
Medications for tremor	2 (0.4%)
CCI, mean (SD)^d^	1.08 (1.39)
Median	0
Minimum, maximum	0.00, 6.00
CCI, n (%)^d^	
0	210 (37.4%)
1	61 (10.9%)
2+	291 (51.8%)
Top 10 diagnoses using ICD-10-GM codes (n, %)^d^	
H52.2: Astigmatism	158 (28.1%)
I10.9: Essential (primary) hypertension not further specified	123 (21.9%)
F32.9: Depressive episode unspecified	122 (21.7%)
G82.4: Spastic tetraplegia	113 (20.1%)
H52.4: Presbyopia	107 (19.0%)
R26.8: Other and unspecified abnormalities of gait and mobility	107 (19.0%)
N31.9: Neuromuscular dysfunction of bladder unspecified	101 (18.0%)
N89.8: Other specified non-inflammatory disorders of vagina^b^	100 (28.4%)
N39.4: Other specified urinary incontinence	99 (17.6%)
G82.1: Spastic paraplegia	98 (17.4%)

*Abbreviations*: *CCI* Charlson Comorbidity Index, *DMT* disease-modifying therapy, *ICD-10-GM* International Classification of Diseases, 10th Revision, German Modification, *IM* intramuscular, *INF* interferon, *MS* multiple sclerosis, *SC* subcutaneous, *SD* standard deviation

^a^More than 1 diagnosis was possible during the index quarter; ^b^calculated only for females

^c^measured in the whole period of 9 quarters (4 quarters pre-index, 1 quarter index, 4 quarters post-index)

^d^measured in the 4 quarters before the index fampridine prescription

### MS-related healthcare resource use and costs before and during fampridine treatment

Regarding the MS-related resource utilization, a high percentage of patients had at least 1 MS-related physical therapy and 1 MS-related outpatient visit during fampridine treatment. One-third of patients had a prescription claim for corticosteroids, and the average number of corticosteroid prescriptions was 0.78 (SD 1.38). Furthermore, 1 in 5 patients had at least 1 day of sick leave due to MS, with a total of 12.6 (SD 45.5) MS-related sick leave days on average.

**Fig. 1:**
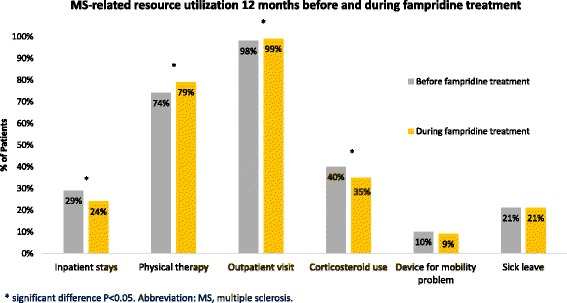
MS-related resource utilization in the 12 months before and during fampridine treatment

Compared to the pre-index period, significant reductions were observed in inpatient stays and corticosteroid use during the fampridine treatment period. The mean number of sick leave days decreased by 2 days, although the difference was not statistically significant (14.7 days [SD 46.8] vs 12.6 days [SD 45.5] *P* = 0.195). The percentage of patients using physical therapy and with outpatient visits increased significantly between the time periods (see Fig. 1).

Data not shown for the pre- and post-index MS-related resource use has been added as supplementary material (Additional file 1).

The overall average number of MS-related outpatient visits was 19 per year (SD 10.4) during the treatment period, implying that 1.6 physician visits per month due to MS were usual for the fampridine-treated patients. The majority of patients had at least 1 MS-related visit at their general practitioner (GP) (7.5 visits on average, SD 7.4). Less than half of the patients visited a neurologist during the fampridine treatment period (4.1 visits on average, SD 5.9).

### MS-related healthcare costs before and during fampridine treatment

After pharmacotherapy, the second highest costs were observed for the inpatient sector. Devices for mobility problems were the smallest cost component, with 0.05% of the total MS-related healthcare costs during the observation period.

Compared with the pre-index period, MS-related inpatient costs declined significantly during fampridine treatment (€1,565.42 vs €1,333.42; *P* < 0.001), whereas MS-related outpatient costs increased significantly during the same period (€518.09 vs €565.47; *P* < 0.0001) (see Table 2).

**Table 2:** MS-related healthcare costs before and during fampridine treatment

	Pre-index period (before fampridine treatment)	Observation period (during fampridine treatment)	*P*-value
	*N* = 562	*N* = 562	
**Inpatient, mean** (SD)	**€1,565.42** (€3,335.18)	**€1,333.42** (€3,882.73)	0.0005
Median	€0	€0	
Minimum, maximum	€0, €30,568.04	€0, €62,415.54	
**Physical therapy, mean** (SD)	**€810.89** (€887.80)	**€963.92** (€925.50)	<0.0001
Median	€613.28	€825.40	
Minimum, maximum	€0, €8,015.80	€0, €6,945.80	
**Outpatient, mean** (SD)	**€518.09** (€341.78)	€**565.47** (€338.85)	<0.0001
Median	€459.33	€508.52	
Minimum, maximum	€0, €2,794.88	€0, €2,851.23	
**Pharmacotherapy**			
**DMTs, mean** (SD)	**€7,684.42** (€8,908.24)	**€8,604.78** (€9,948.43)	<0.0001
Median	€0	€0	
Minimum, maximum	€0, €29,157.08	€0, €33,639.54	
**Corticosteroids, mean** (SD)	**€108.24** (€194.47)	**€88.88** (€181.15)	0.0054
Median	€0	€0	
Minimum, maximum	€0, €989.38	€0, €1,041.33	
**Fampridine, mean (**SD**)**	**€0** (€0)	**€5,519.32** (€1,565.83)	<0.0001
Median	€0	€5,908.53	
Minimum, maximum	€0, €0	€225.11, €10,033.99	
**Other MS-related prescriptions, mean** (SD)	**€267.10** (€525.92)	**€306.90** (€642.63)	0.1229
Median	€52.16	€55.53	
Minimum, maximum	€0, €4,782.96	€0, €7,358.06	
**Devices for mobility problems, mean** (SD)	**€6.09** (€26.95)	**€9.17** (€58.20)	0.7468
Median	€0	€0	
Minimum, maximum	€0, €344.01	€0, €1,146.39	
**Total MS-related healthcare, mean** (SD)	**€10,960.26** (€9,030.32)	**€17,391.86** (€10,325.65)	<0.0001
Median	€9,376.59	€14,447.76	
Minimum, maximum	€0, €44,126.80	€1,107.41, €67,001.71	

Bolded text indicates the main message – the mean values and the categories

*Abbreviations*: *DMT* disease-modifying therapy, *MS* multiple sclerosis, *SD* standard deviation

### Stratified analyses

**Fig. 2:**
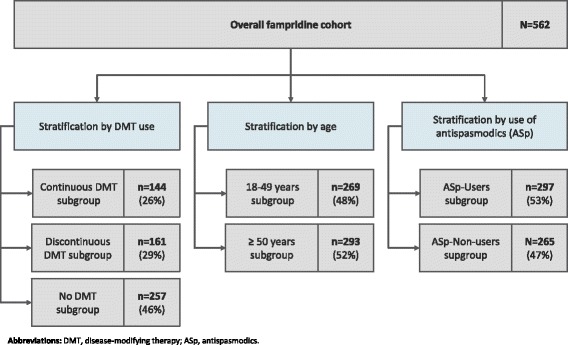
Subgroups

About one-quarter of the identified patients had continuous DMT treatment and most (46%) did not use any DMT during the whole study period. Just under one-half (48%) of patients were younger than 50 years of age and more than half (53%) used antispasmodics at least once (53%) (see Fig. 2).

## DMT stratification

Overall, a greater proportion of patients (46%) had no evidence of DMT treatment during the full observation period, compared with discontinuous DMT use (29%), and continuous use (26%). These subgroups differed in age, comorbidities, MS-related inpatient stays and costs, and MS-related sick leave.

Concerning the inpatient stays, those with discontinuous DMT use had the highest proportion with MS-related hospitalizations (30%) in contrast to the no DMT (27%) and the continuous DMT (10%) subgroups. However, the decline in MS-related stays from the pre- to post-index period was the highest and only significant in the no DMT subgroup (35–27%, *P* = 0.007).

In addition, the mean number of sick leave days and corticosteroid prescriptions declined significantly during the fampridine treatment period within the no DMT cohort (MS-related sick leave days: mean, 12.0–5.7 days, *P* = 0.002; corticosteroid prescriptions: 0.9–0.7, *P* = 0.013).

The inpatient costs declined significantly from the pre- to the post-index period in the no DMT subgroup (€2,004 vs €1,600, *P* < 0.001), whereas no significant differences could be observed within the other subgroups (€458 vs €457, *P* = 0.872 continuous DMT subgroup; €1,856 vs €1,691, *P* = 0.174 discontinuous DMT subgroup). Partly due to the lack of DMT costs, the no DMT subgroup had the lowest MS-related healthcare costs (€9,197), with €26,984 in the continuous DMT subgroup and €21,893 in the discontinuous DMT subgroup.

## Stratification by age

Almost half of the patients were younger than 50 years (48%), and older patients had a higher CCI than younger patients (1.81 vs 1.06). Over half (57%) of the older and one-third (34%) of the younger age subgroups did not use DMTs. During fampridine treatment, 27% of the younger subgroup and 21% of the older subgroup had MS-related inpatient stays. These rates of MS-related hospitalization were significantly lower than in the pre-index period, with 33% in the younger age subgroup (*P* < 0.05) and 26% in the older age subgroup (*P* < 0.05) hospitalized.

Total MS-related healthcare costs in the treatment period for those aged ≥50 years were €14,920, and €20,804 for those aged 18 to 49 years. The second highest cost component next to pharmacotherapy was the inpatient sector among the younger aged subgroup and physical therapy in the older aged subgroup.

## Stratification by antispasmodic treatment

**Fig. 3:**
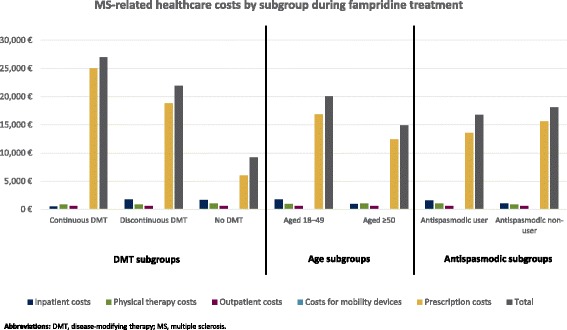
MS-related healthcare costs by subgroup during fampridine treatment

Fifty-three percent (*n* = 297) of the fampridine patients had at least 1 prescription claim for antispasmodics during the study period. Twenty-seven percent of these had MS-related inpatient stays. Among the antispasmodic non-users, 20% were hospitalized due to MS in the post-index period. In the pre-index period, the MS-related hospitalizations were significantly higher, with 33% (*P* < 0.05) and 26% (*P* < 0.05) compared to the post-index period for the users and non-users, respectively. The MS-related total costs were €18,100 in the antispasmodic non-user subgroup and €16,760 in the antispasmodic user subgroup (see Fig. 3).

## Discussion

This study shows the differences in MS-related healthcare resource use and costs of patients in Germany initiating and continuing treatment with fampridine for at least 12 months compared to the 12 months prior to treatment initiation.

Patients starting fampridine treatment were, on average, 50 years old, which demonstrates that the disease had already progressed, as the average age for disease onset is 30 [12]. The mean age at the start of fampridine therapy, however, is slightly lower in Germany than in the United States (US), where the mean age is 55 years [13].

Before commencing fampridine treatment, many patients used medications such as muscle relaxants and antidepressants, which is similar to other findings [13]. The percentage of non-DMT users was slightly higher with 46% in this German population compared to 38% of MS patients in the US, as noted by M Jara, MF Sidovar and HR Henney [13] in 2014. Almost every patient had at least 1 outpatient visit, 24% were hospitalized due to MS in the treatment period, and 81% utilized physical therapy in addition to fampridine treatment. This study reveals that the combination of fampridine treatment and physical therapy is common in Germany, supporting the fact that fampridine is used complementary to rather than in place of physical therapy [14, 15] whereas physical therapy was deemed the appropriate comparator in the fampridine German Arzneimittelmarkt-Neuordnungsgesetz AMNOG) (ie, evaluation of new pharmaceuticals in Germany) value dossier. However, as the requested comparison did not include sufficient data, no additional benefit was stated [14]. The significant reductions in corticosteroid use and inpatient stays after initiating fampridine might be due to improvement of mobility problems. Improvement could also be due to the increase in physical therapy. Furthermore, the increasing use of physical therapy might also suggest that patients became more active to deal with mobility issues after experiencing the benefit from fampridine. It is also possible that individuals motivated to initiate and adhere to fampridine treatment might also be subsequently motivated to attend physical therapy sessions. In addition to physical therapy, other outpatient care played an important role in treating MS (approximately 19 visits per year per patient), as GPs were contacted at least twice and neurologists at least once per quarter. M Jara, MF Sidovar and HR Henney [13] reported that 79.1% of the first fampridine prescriptions were prescribed by neurologists in the US, which is higher than the estimated 45% of MS patients visiting a neurologist for their MS in our study.

The total MS-related healthcare costs were significantly higher in the fampridine treatment period compared to the period before fampridine treatment, mainly due to the increased pharmacotherapy costs. Pharmacotherapy accounted for 82% of post-index MS-related costs, followed by the inpatient sector, with 8%. A high percentage of prescription costs relative to overall MS-related costs (65%) was also found by JD Prescott, S Factor, M Pill and GW Levi [16] in 2004. However, in contrast to the increasing pharmacotherapy costs, the MS-related inpatient costs declined during fampridine treatment compared to the pre-treatment period (€1,333 vs €1,565, *P* < 0.001). This means that while the main cost driver (pharmacotherapy) increased, the second highest cost component (inpatient costs) declined simultaneously.

The different patient subgroup analyses revealed findings that were consistent with the overall analysis. Prescription costs were the highest in all subgroups, followed by inpatient costs, except within the continuous DMT and ≥50-year-old subgroups, where physical therapy costs were higher than the inpatient costs. However, slight differences were observed, for example in the 3 subgroups measuring DMT treatment concerning characteristics such as age, comorbidity burden, and MS-related inpatient stays. The no DMT subgroup mostly had significant changes from pre- to post-fampridine initiation, including MS-related hospitalizations, corticosteroid use, and MS-related sick leave days. It was assumed that these patients were not relapsing-remitting MS patients; therefore, they had limited options for DMT treatment and may benefit the most from fampridine. Another explanation might be that without DMT treatment these patients were more willing to adhere to fampridine treatment and subsequently also physical therapy.

Several limitations of this study should be mentioned. First, there were no comparisons to fampridine discontinuers or non-users, and further research is warranted in these areas as the results cannot be generalized to those patient groups. Second, no information on clinical outcomes, such as Expanded Disability Status Scale scores, is available in claims data, so the severity of the disability could not be evaluated. Third, no adjustments, such as for the use of physical therapy, outpatient visits, or disease progression, were made and therefore the impact of these aspects on the outcomes could not be estimated. Fourth, claims data are not collected for research but instead for accounting purposes and therefore include only sectors that are reimbursed by the statutory health insurance. Therefore, indirect costs such as societal costs of MS-attributable informal care could not be assessed. Additionally, compliance with medication regimens could only be approximated based on prescription fills, as the actual intake is not observable in this data source. Last, the number of outpatient visits could only be approximated and may be underestimated, as flat charges for outpatient visits on a quarterly basis exist in Germany.

## Conclusion

This study provides insights into the treatment of MS patients in Germany beginning treatment with fampridine and continuing treatment for at least 12 months. These patients visit the GP and neurologist regularly, and physical therapy is used in combination with fampridine treatment in almost every case. Besides the pharmacotherapy costs, the inpatient costs were the second most important cost driver in all but 2 patient subgroups. Inpatient stays, as well as the costs, declined during fampridine treatment compared to the pre-treatment period. The overall costs, however, increased due to the pharmaceutical costs. This cost increase might be justified due to improved patient outcomes beyond the reduced healthcare utilization; however, patient reported outcomes are not available within the Statutory Health Insurance. To better understand fampridine influence in the real world, further research is necessary.

## Additional file

Additional file 1: MS-related healthcare resource use before and during fampridine treatment. Description of data: The additional file includes an overview of the MS-related healthcare resource use in the pre- and post-index period in a tabular format. (DOCX 17 kb)

## Abbreviations

AMNOG: Arzneimittelmarkt-Neuordnungsgesetz
ASp: Antispasmodics
ATC: Anatomical therapeutic chemical classification system
CCI: Charlson Comorbidity Index
DMT: Disease-modifying therapy
GP: General practitioner
HRI: Health Risk Institute
ICD-10-GM: International Classification of Diseases, 10th Revision, German Modification
IM: Intramuscular
INF: Interferon
MS: Multiple sclerosis
SC: Subcutaneous
SD: Standard deviation
US: United States
