# Supplementary material for: Healthcare resource use and costs of multiple sclerosis patients in Germany before and during fampridine treatment
Source: BMC Neurol. 2017 Mar 27;17:62. doi: 10.1186/s12883-017-0844-z (PMC5369011; doi:10.1186/s12883-017-0844-z)
Supplement: Additional file 1: — MS-related healthcare resource use before and during fampridine treatment. Description of data: The additional file includes an overview of the MS-related healthcare resource use in the pre- and post-index period in a tabular format. (DOCX 17 kb) [file 12883_2017_844_MOESM1_ESM.docx]

Additional file 1

MS-related healthcare resource use before and during fampridine treatment

|  | **Pre-index period (before fampridine treatment)**  **N=562** | **Observation period (during fampridine treatment)**  **N=562** | ***P*-value** |
| --- | --- | --- | --- |
| **Inpatient sector** |  |  |  |
| **Any hospitalization, n** (%) | 165 (29.4%) | 133 (23.7%) | 0.0020 |
| **Number of hospitalizations, mean** (SD)  Median  Minimum, maximum | 0.68 (1.45)  0  0.22, 13.00 | 0.52 (1.22)  0  0.00, 11.00 | 0.0010 |
| **Total length of stay in days, mean (**SD**)**  Median  Minimum, maximum | 4.57 (14.06)  0  0.00, 276.00 | 3.90 (15.49)  0  0.00, 276.00 | 0.0005 |
| **Physical therapy**  **Any physical therapy, n** (%) | 416 (74.0%) | 443 (78.8%) | 0.0050 |
| **Number of physical therapy sessions, mean** (SD)  Median  Minimum, maximum | 3.60 (3.58)  3  0.00, 30.00 | 4.16 (3.71)  4  0.00, 26.00 | <0.0001 |
| **Outpatient sector**  **Any outpatient visit, n** (%) | 550 (97.9%) | 557 (99.1%) | 0.0350 |
| **Estimated number of outpatient visits, mean** (SD)  Median  Minimum, maximum | 17.23 (10.64)  16  0.00, 72.00 | 18.92 (10.39)  17  0.00, 54.00 | <0.0001 |
| **Pharmacotherapy sector**  **Corticosteroid use**  **Number of corticosteroid prescriptions, mean** (SD)  Median  Minimum, maximum | 225 (40.0%)  0.96 (1.53)  0  0.00, 10.00 | 196 (34.9%)  0.78 (1.38)  0  0.00, 8.00 | 0.0270  0.0019 |
| **Number of fampridine prescriptions, mean** (SD)  Median  Minimum, maximum | 1. (0.00)   0  0.00, 0.00 | 11.19 (3.39)  12  1.00, 22.00 | <0.0001 |
| **Any device for mobility problems, n** (%) | 55 (9.8%) | 50 (8.9%) | 0.5920 |
| **Any sick leave day, n** (%) | 118 (21.0%) | 115 (20.5%) | 0.7100 |
| **Number of sick leave days, mean** (SD)  Median  Minimum, maximum | 14.69 (46.76)  0  0.00, 338.00 | 12.64 (45.50)  0  0.00, 366.00 | 0.1954 |

**Abbreviations:** DMT, disease-modifying therapy; MS, multiple sclerosis; SD, standard deviation.
